# Biological Effects of an Isoquercetin-Containing Formulation in Preclinical Models of Skin Aging

**DOI:** 10.7759/cureus.104733

**Published:** 2026-03-05

**Authors:** Rossana Vasconcelos, Milena Barrera Silva, Tomaz José Aquino Vasconcelos do Carmo, Letícia A de Santis, Elisete I Crocco

**Affiliations:** 1 Dermatology, Universtity of Santo Amaro, São Paulo, BRA; 2 Dermatology, University of Santo Amaro, São Paulo, BRA; 3 Dermatology, Irmandade Santa Casa de Misericórdia de São Paulo, São Paulo, BRA

**Keywords:** compound containing isoquercetin, experimental dermatology, glycation, human skin explants, inflammaging, nrf2 pathway, oxidative stress, photoaginge, preclinical study, skin aging

## Abstract

Background

Skin aging involves oxidative stress, inflammation, extracellular matrix degradation, and glycation. Bioactive compounds targeting multiple pathways may offer mechanistic advantages in skin aging modulation.

Objective

This study aimed to evaluate the biological effects of a compound containing isoquercetin using preclinical in vitro and ex vivo skin models.

Methods

Five independent assays were conducted in human skin explants, reconstructed human epidermis enriched with peripheral blood mononuclear cells (PBMCs), and human dermal fibroblasts (HFF-1). NQO1 gene expression was quantified by quantitative real-time polymerase chain reaction (qRT-PCR). IL-6 and IL-8 levels were measured using cytometric bead array. UV radiation-induced oxidative stress was assessed by radical protein assays. Ki-67 and type I pro-collagen expression were evaluated by immunofluorescence. Antiglycation and deglycation activity were assessed through carboxymethyl-lysine (CML) quantification. Data were analyzed using Student’s t-test or one-way ANOVA.

Results

The compound significantly increased NQO1 expression (+78%, p < 0.05) in human skin explants. In the inflammatory model, IL-6 and IL-8 levels were reduced by 45% and 50%, respectively (p < 0.001). UV radiation-induced oxidative protein markers were reduced (radical protection: 66.79%, p < 0.001). Ki-67 expression increased by 41.83% (p < 0.05), and type I pro-collagen synthesis increased 2.01-fold (p < 0.0001). In fibroblast models, antiglycation and deglycation assays demonstrated reductions in CML levels of 56.90% and 39.75%, respectively (p < 0.001).

Conclusion

In preclinical skin models, a compound containing isoquercetin significantly modulated oxidative stress, inflammatory response, collagen synthesis, and glycation pathways. Controlled clinical studies are required to determine whether these biological effects translate into measurable outcomes in human subjects.

## Introduction

Skin aging is widely recognized as a multifactorial biological process arising from the dynamic interaction between intrinsic factors related to chronological aging and extrinsic influences such as UV radiation exposure, pollution, and other environmental stressors [1,2]. These factors contribute to structural and functional alterations of the skin, including extracellular matrix degradation, chronic low-grade inflammation, and reduced endogenous antioxidant capacity.

Among the mechanisms most consistently implicated in skin aging, oxidative stress and glycation play central roles. UV radiation-induced production of reactive oxygen species promotes cumulative cellular damage and contributes to matrix degradation pathways involved in skin aging [1,2]. In parallel, non-enzymatic glycation of dermal proteins leads to the formation of advanced glycation end products, which further compromise skin structure and function. These processes are considered important targets for preventive and therapeutic strategies in dermatology and cosmetic science [2,3].

Naturally derived bioactive compounds have gained increasing attention due to their capacity to modulate multiple biological pathways simultaneously. Isoquercetin, a glycosylated flavonoid derived from quercetin, has been investigated for its antioxidant and anti-inflammatory properties. In the present study, isoquercetin was incorporated as one of the active components of a topical formulation that also contained microencapsulated retinol, vitamin E, two molecular weights of hyaluronic acid, and nicotinamide. These ingredients were selected based on their complementary antioxidant, regenerative, barrier-supporting, and anti-inflammatory characteristics [4-7].

Despite growing interest in flavonoid-based formulations, the literature still lacks integrated preclinical investigations evaluating the biological activity of isoquercetin-containing formulations across multiple cellular and histological parameters associated with skin aging.

Therefore, this exploratory preclinical study aimed to investigate the biological activity of an isoquercetin-containing formulation in validated preclinical models of skin aging.

## Materials and methods

Study design

This exploratory preclinical experimental study was conducted to investigate the biological activity of an isoquercetin-containing formulation using validated in vitro and ex vivo skin models. Five complementary experimental assays were performed to evaluate antioxidant, anti-inflammatory, regenerative, and antiglycation/deglycation effects.

All experiments were conducted under controlled laboratory conditions following predefined protocols. Treatment allocation was determined according to the experimental design prior to assay initiation.

Due to the mechanistic and exploratory nature of the study, full operator blinding during treatment application was not implemented. However, quantitative measurements, including quantitative real-time polymerase chain reaction (qRT-PCR), cytokine immunoassays, oxidative stress assays, and image-based fluorescence quantification, were performed using standardized laboratory analytical systems to reduce operator-dependent variability.

Biological replicates were included according to the experimental model. Where applicable, measurements from explants derived from the same donor were averaged prior to intergroup statistical comparison to minimize pseudoreplication.

Biological materials and experimental models

Human Skin Explants

Human skin explants were obtained from adult donors undergoing elective surgical procedures, in compliance with institutional ethical standards. Samples were anonymized prior to laboratory processing.

Inclusion criteria required clinically healthy skin without active inflammatory dermatoses, infection, or recent use of topical or systemic retinoids. Samples presenting macroscopic abnormalities or compromised structural integrity were excluded.

Explants used for NQO1 gene expression, oxidative stress assessment, and immunofluorescence analyses were derived from two adult donors (50 and 61 years old) and processed under standardized conditions immediately after collection.

Human Dermal Fibroblasts (HFF-1)

The HFF-1 human dermal fibroblast cell line was used for antiglycation and deglycation assays. Cells were cultured under standard conditions (37°C, 5% carbon dioxide (CO₂)) and maintained at comparable passage numbers to reduce variability.

Three-Dimensional Reconstructed Epidermis with Peripheral Blood Mononuclear Cells (PBMCs)

Three-dimensional reconstructed human epidermis models enriched with PBMCs were used to evaluate inflammatory cytokine modulation. PBMCs were obtained from healthy adult donors and pooled prior to co-culture to reduce donor-specific variability. The co-culture system was selected to model epithelial-immune interactions relevant to cutaneous inflammatory processes.

Tested formulation

The tested formulation contained isoquercetin, microencapsulated retinol, vitamin E, hyaluronic acid in two molecular weights, and nicotinamide. The formulation was incorporated into a dermatologically tested topical emulsion system optimized for in vitro application.

Exact concentrations of individual components cannot be disclosed due to proprietary and intellectual property considerations. All ingredients were incorporated within internationally accepted cosmetic safety ranges, and all active ingredients were sourced from certified suppliers and met cosmetic-grade purity standards.

The incorporation of flavonoids into optimized topical delivery systems has been previously described as an approach to enhance stability, solubility, and skin permeation in cutaneous applications [8,9].

Application parameters

The formulation was applied under standardized experimental conditions across assays, with a defined exposure duration of 48 hours for explant-based assays unless otherwise specified. Vehicle-only controls were included when applicable.

Antioxidant Activity: NQO1 Gene Expression

Skin explants were treated for 48 hours. Total RNA extraction, cDNA synthesis, and qRT-PCR were performed using standardized laboratory procedures.

Relative NQO1 gene expression was calculated using the ΔΔCt method with GAPDH as the endogenous control gene. Amplification efficiency was validated prior to analysis and remained within acceptable ranges (90%-110%). Primer performance and amplification specificity were confirmed according to standard laboratory quality control procedures, and negative controls were included in all runs to confirm the absence of contamination and PCR inhibitors.

Quadruplicate explants per donor were analyzed, and measurements were averaged per donor before statistical comparison.

Inflammatory Cytokine Analysis (IL-6 and IL-8)

Reconstructed epidermal models enriched with PBMCs were stimulated with lectin (2.5 µg/mL). Experimental groups included basal control, lectin-stimulated control, lectin combined with dexamethasone (1 µM) as a positive control, and lectin combined with the isoquercetin-containing formulation.

IL-6 and IL-8 concentrations were quantified using cytometric bead array (CBA) methodology according to manufacturer instructions. Experiments were conducted in triplicate, with independent runs.

Oxidative Stress Assessment

Skin explants were exposed to UV radiation (10 J/cm²) to induce oxidative stress, while non-irradiated explants served as negative controls. Radical status factor (RSF) and radical protection (RP) were determined using immunoenzymatic assays based on quantification of radicalized proteins according to standardized laboratory procedures.

Experiments were conducted in triplicate, with independent runs.

Immunofluorescence Analysis: Ki-67 and Type I Pro-collagen

Immunofluorescence staining was performed to evaluate epidermal proliferation (Ki-67) and type I pro-collagen expression. Twelve microscopic fields per explant were analyzed using a standardized scanning approach across the tissue section, and image acquisition parameters were maintained consistently across samples.

Quantification was performed using standardized image analysis software according to laboratory procedures. Fluorescence intensity was normalized to tissue area and background signal using predefined analytical thresholds. Sections presenting evident processing artifacts were excluded from analysis.

Quadruplicate explants per donor were evaluated.

Antiglycation and Deglycation Assays

HFF-1 fibroblasts were exposed to glyoxal (200 µM) to induce glycation. This concentration was selected based on established in vitro glycation models demonstrating reliable induction of advanced glycation end products while preserving cell viability.

Two experimental protocols were conducted, consisting of antiglycation treatment administered prior to glyoxal exposure and deglycation treatment applied after glycation establishment. Carboxymethyl-lysine (CML) levels were quantified using immunoenzymatic assays.

Experiments were conducted in triplicate, with independent runs.

Statistical analysis

Statistical analyses were performed according to predefined laboratory protocols. Data are presented as mean ± standard error of the mean (SEM) or mean ± SD, as reported in the original laboratory output for each assay.

Comparisons between two groups were analyzed using an unpaired two-tailed Student’s t-test, while comparisons involving three or more groups were analyzed using one-way ANOVA as a global test. When statistically significant, post hoc analyses were conducted according to the predefined laboratory methodology, with Tukey’s post hoc test applied in assays requiring multiple pairwise comparisons and Fisher’s least significant difference (LSD) test used in assays where it was specified as the predefined analytical method.

Raw replicate-level datasets were not available for independent reassessment of distributional assumptions or recalculation of alternative statistical models. This limitation is acknowledged as part of the exploratory preclinical design. A p-value < 0.05 was considered statistically significant.

## Results

NQO1 gene expression analysis

Treatment of human skin explants with the isoquercetin-containing formulation was associated with increased NQO1 gene expression compared with untreated controls. Skin explants from two donors (50 and 61 years old) were analyzed, with experiments performed in quadruplicate (n = 4 per condition, averaged per donor prior to statistical comparison).

**Figure 1:**
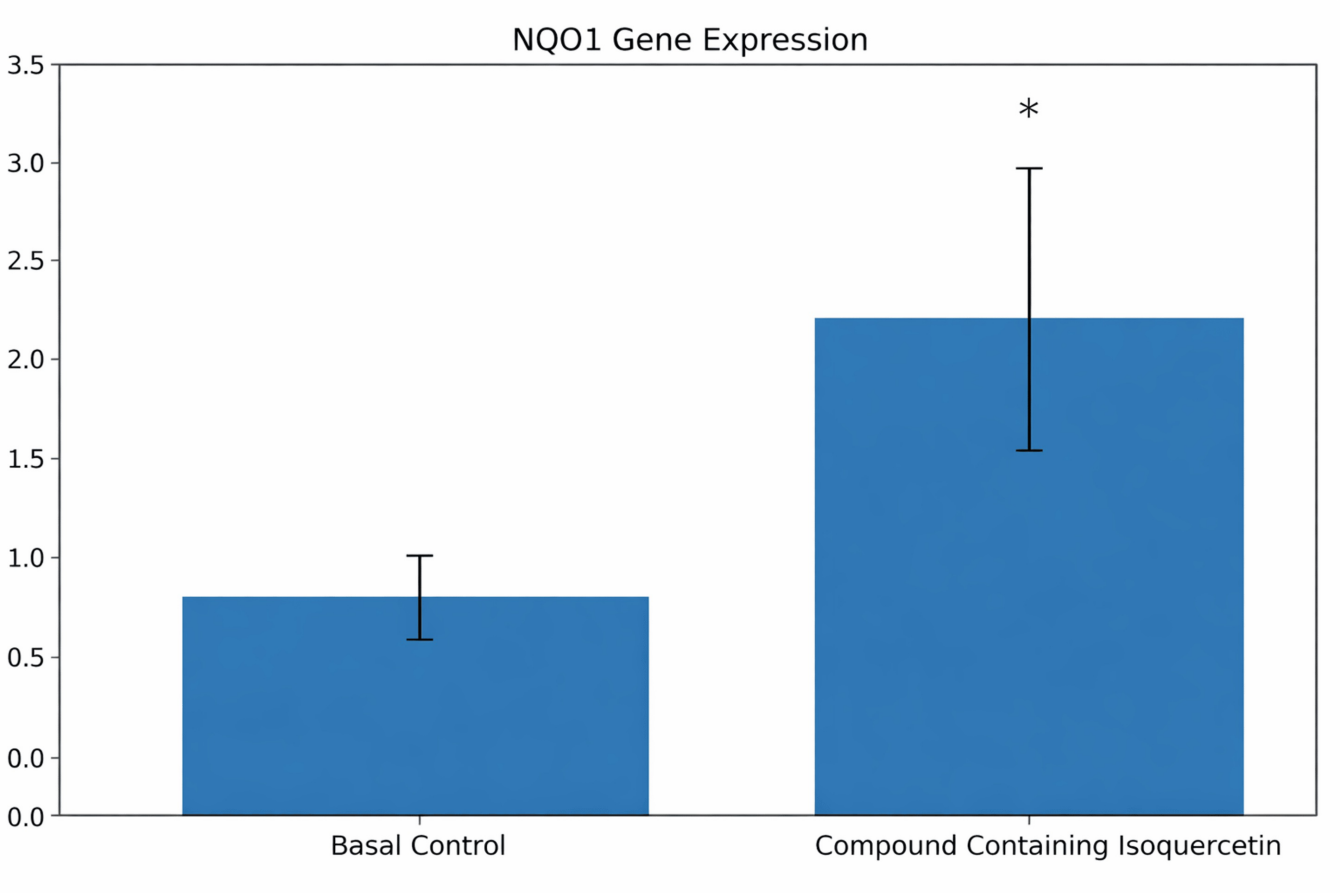
Relative NQO1 gene expression in human skin explants treated with an isoquercetin-containing formulation Values are expressed as mean ± standard error of the mean (SEM) relative to the basal control. Experiments were performed in quadruplicate (n = 4 per condition). Statistical analysis was conducted using one-way ANOVA followed by Fisher’s least significant difference (LSD) post hoc test. A p-value < 0.05 was considered statistically significant. *p < 0.05 versus basal control.

qRT-PCR demonstrated a statistically significant increase in NQO1 expression after 48 hours of treatment (one-way ANOVA followed by Fisher’s LSD post hoc test, p < 0.05) (Figure 1). Under the experimental conditions evaluated, these findings are consistent with activation of endogenous antioxidant response pathways within the ex vivo model.

Anti-inflammatory activity: IL-6 and IL-8 modulation

In the three-dimensional human epidermal model co-cultured with PBMCs, treatment with the isoquercetin-containing formulation was associated with reduced secretion of the pro-inflammatory cytokines IL-6 and IL-8 following lectin stimulation. Experiments were conducted in triplicate independent runs (n = 3 per group).

**Figure 2:**
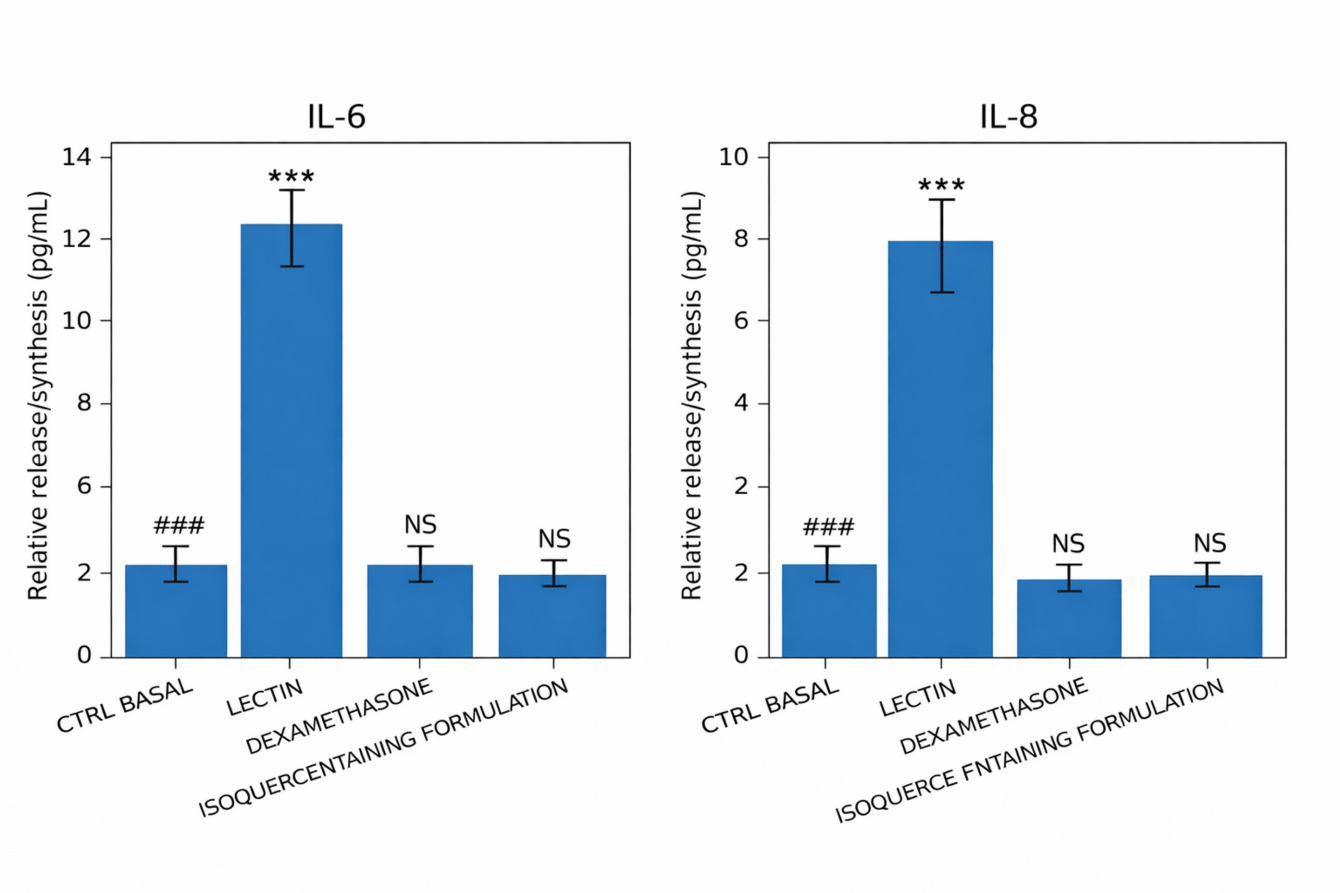
Cytometric bead array (CBA) immunoassay for the quantification of inflammatory cytokines in a keratinocyte-based three-dimensional reconstructed epidermal model co-cultured with peripheral blood mononuclear cells (PBMCs). Cells were treated with lectin (2.5 µg/mL), dexamethasone (1 µM), or an isoquercetin-containing formulation. (A) Relative synthesis/release of IL-6. (B) Relative synthesis/release of interleukin-8 (IL-8). Values are expressed as mean ± standard error of the mean (SEM) of relative protein levels. Experiments were performed in triplicate (n = 3 per group) according to the laboratory protocol. Statistical analysis was conducted using one-way ANOVA followed by Fisher’s least significant difference (LSD) post hoc test. A p-value < 0.05 was considered statistically significant. ***p < 0.001 versus lectin-treated group; ###p < 0.001 versus basal control (CTRL) group; NS, not significant.

Compared with lectin-stimulated controls, treatment resulted in statistically significant reductions in IL-6 and IL-8 concentrations (one-way ANOVA followed by Fisher’s LSD post hoc test, p < 0.001) (Figures 2A, 2B). No statistically significant difference was observed between the treatment group and the dexamethasone-treated group under the experimental conditions tested.

These findings indicate anti-inflammatory activity within the defined in vitro co-culture system.

Oxidative stress: RSF and RP

**Figure 3:**
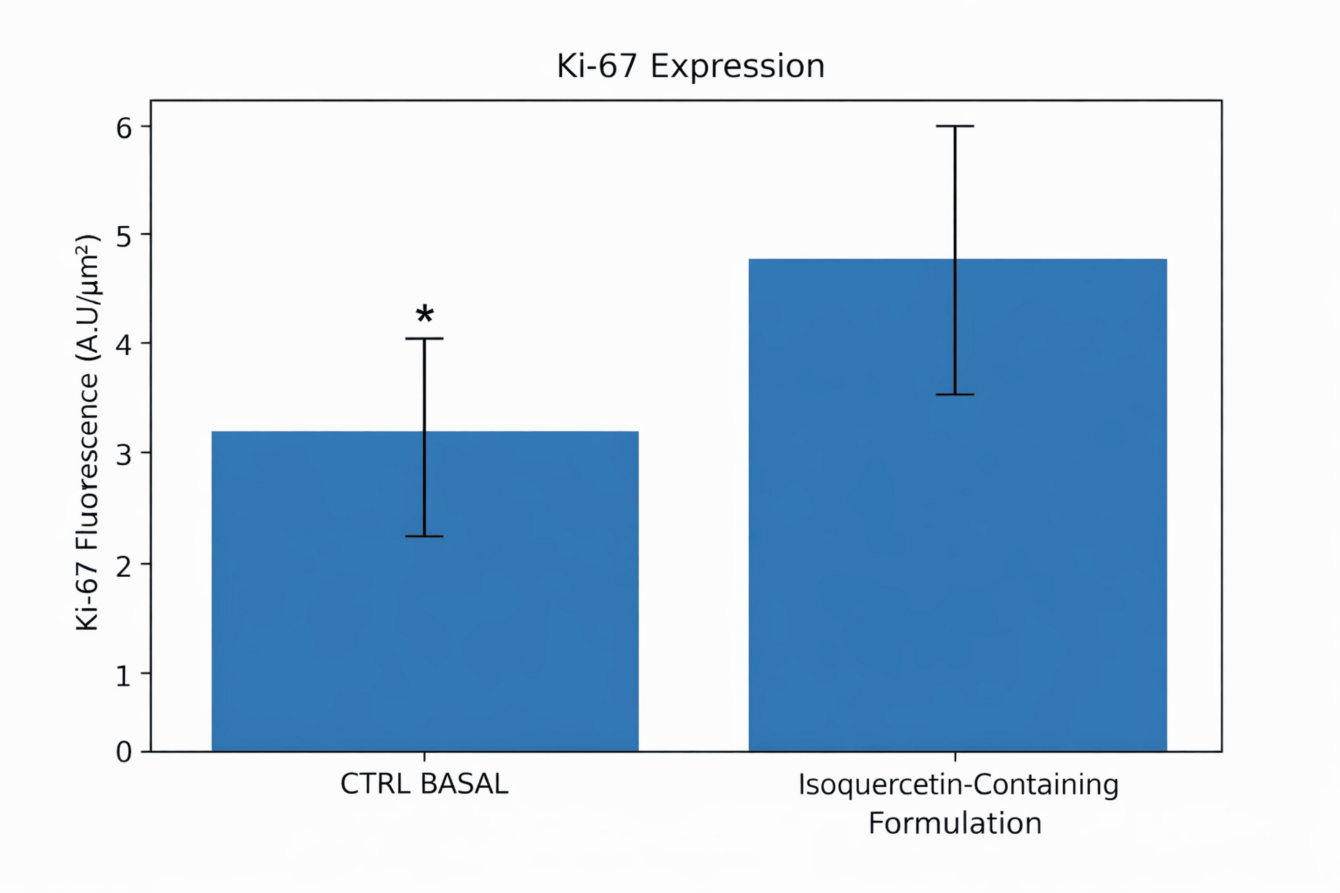
Semi-quantitative analysis of Ki-67 immunofluorescence intensity in human skin explants treated with an isoquercetin-containing formulation Data are expressed as mean ± standard deviation (SD). Twelve microscopic fields per explant were analyzed, and quadruplicate explants per donor were evaluated. Statistical analysis was performed using an unpaired two-tailed Student’s t-test. *p < 0.05 versus basal control (CTRL) group.

Exposure of human skin explants to UV radiation (10 J/cm²) resulted in a 2.27-fold increase in radicalized protein formation compared with non-irradiated controls. Treatment with the isoquercetin-containing formulation was associated with a statistically significant reduction in UV radiation-induced protein radicalization (n = 3 independent runs; one-way ANOVA with Tukey post hoc test, p < 0.001) (Figure 3).

Based on these data, an RSF of 3.01 was calculated, corresponding to an RP value of 66.79%. Within the context of the UV-induced oxidative stress model employed, these findings are consistent with antioxidant activity under controlled experimental conditions.

Epidermal renewal and dermal matrix markers

Ki-67 Expression

**Figure 4:**
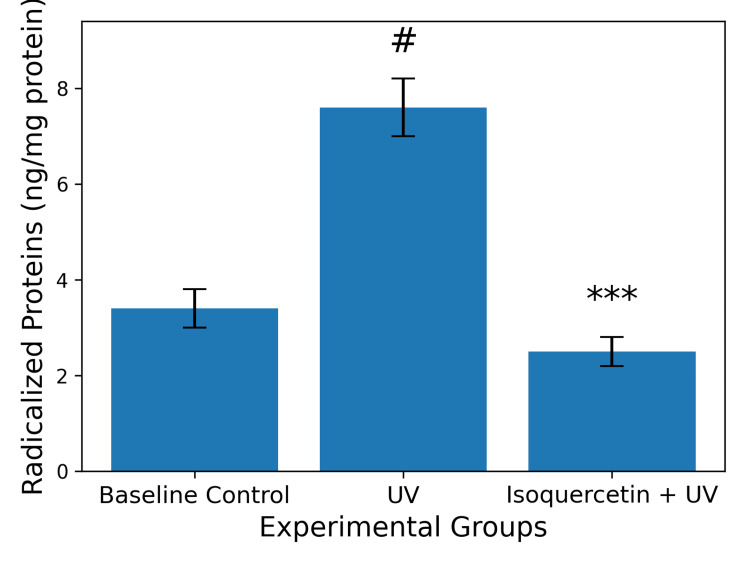
Radicalized protein synthesis in human skin explant cultures exposed to ultraviolet (UV) radiation (10 J/cm²). Data are expressed as mean ± standard deviation (SD) from three replicates (n = 3), according to the laboratory protocol. Non-irradiated explants served as basal controls. Statistical analysis was performed using one-way ANOVA followed by Tukey’s post hoc test. #p < 0.001 versus basal control group; ***p < 0.001 versus UV-exposed group.

**Figure 5:**
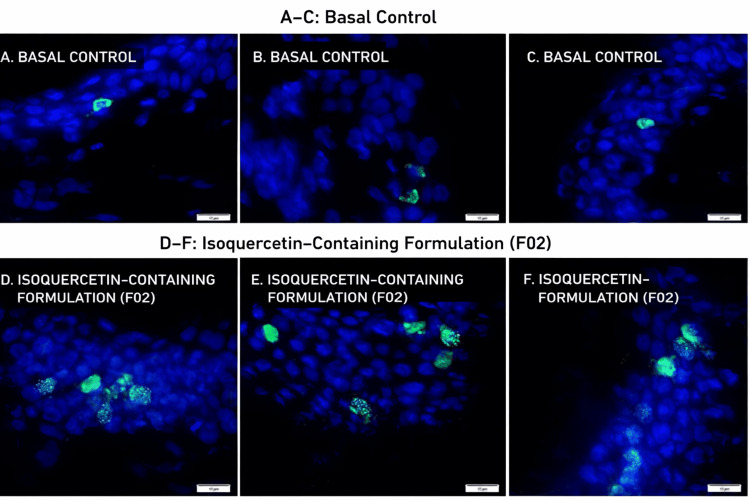
Immunofluorescence analysis of Ki-67 protein expression in human skin explants treated with an isoquercetin-containing formulation (FORMULA F02). A–C: Histological sections of ex vivo human skin without treatment (basal control); D–F: Histological sections of ex vivo human skin treated with the isoquercetin-containing formulation (FORMULA F02); Ki-67 protein is shown in green, and cell nuclei (DNA) are counterstained in blue. Scale bar = 10 µm.

Immunofluorescence analysis demonstrated increased Ki-67 expression in treated explants compared with untreated controls. Semi-quantitative fluorescence analysis across 12 evaluated microscopic fields per explant showed a 41.83% increase in Ki-67 expression (unpaired Student’s t-test, p < 0.05) (Figures 4, 5).

Within the limitations of the ex vivo model and replication structure, these findings are consistent with increased epidermal proliferative activity under the experimental conditions tested.

Type I Pro-Collagen Expression

**Figure 6:**
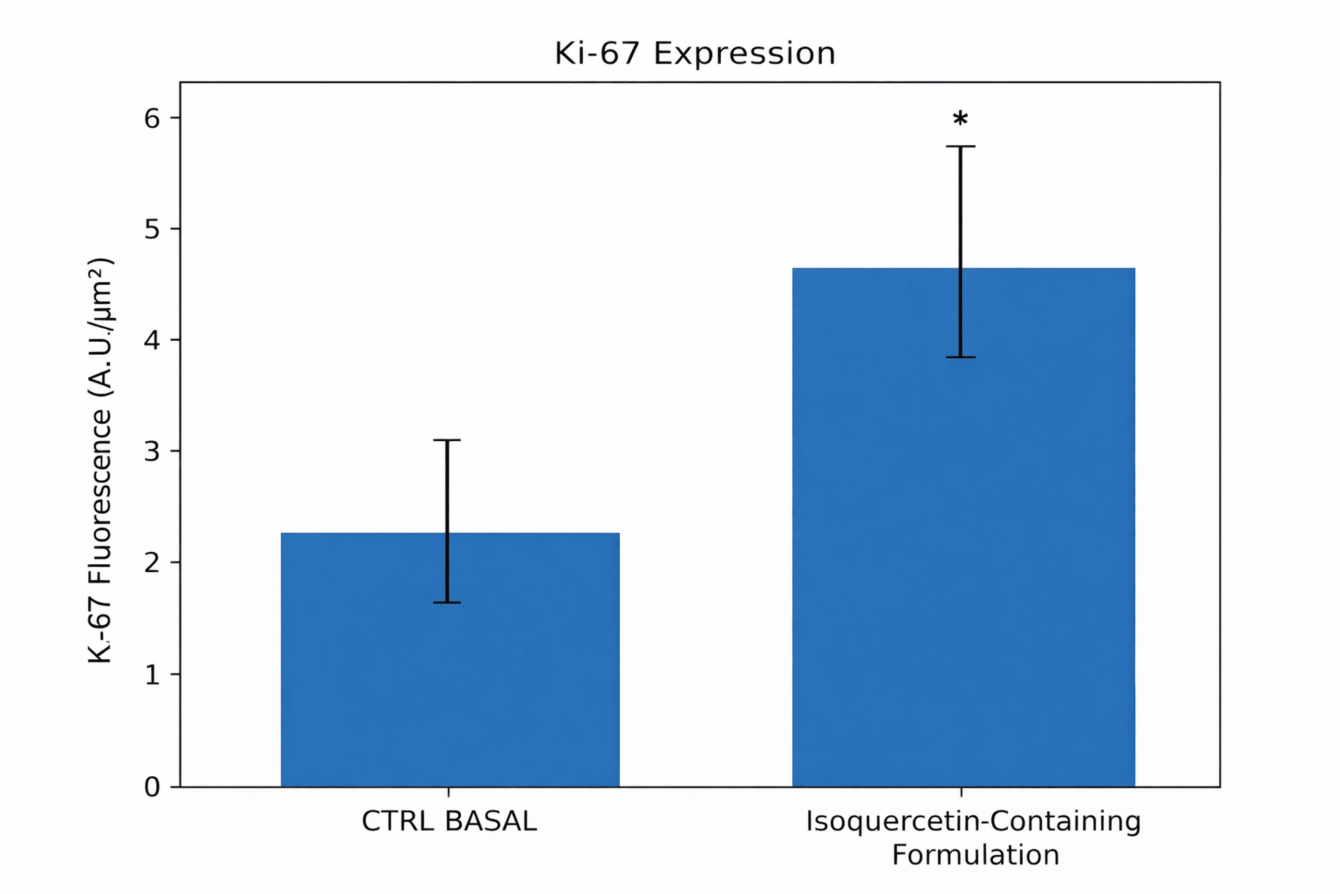
Semi-quantitative analysis of type I pro-collagen immunofluorescence intensity in human skin explants treated with an isoquercetin-containing formulation. Data are expressed as mean ± standard deviation (SD) from 12 analyzed microscopic fields. Statistical analysis was performed using Student’s t-test. *p < 0.0001 versus basal control (CTRL) group.

**Figure 7:**
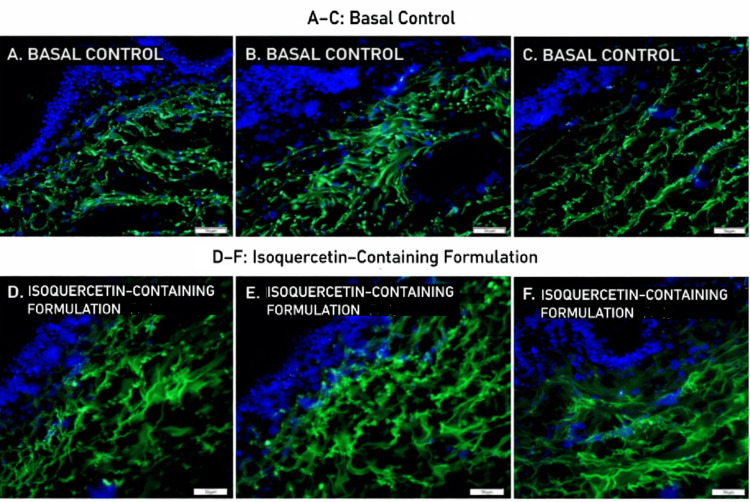
Immunofluorescence evaluation of type I pro-collagen expression in human skin explants treated with an isoquercetin-containing formulation. (A–C) Representative histological sections of ex vivo human skin without treatment (basal control); (D–F) Representative histological sections of ex vivo human skin treated with an isoquercetin-containing formulation; Type I pro-collagen is shown in green, and cell nuclei (DNA) are counterstained in blue. Scale bar = 50 µm.

Treatment with the isoquercetin-containing formulation was associated with a 2.01-fold increase in type I pro-collagen expression compared with control samples (unpaired Student’s t-test, p < 0.0001) (Figure 6 and Figure 7).

Within the defined ex vivo experimental framework, these results suggest modulation of extracellular matrix-related protein expression.

Antiglycation and Deglycation Activity

In HFF-1 fibroblast cultures exposed to glyoxal (200 µM), the isoquercetin-containing formulation was associated with reductions in CML levels under both preventive and reversal protocols (n = 3 per group).

**Figure 8:**
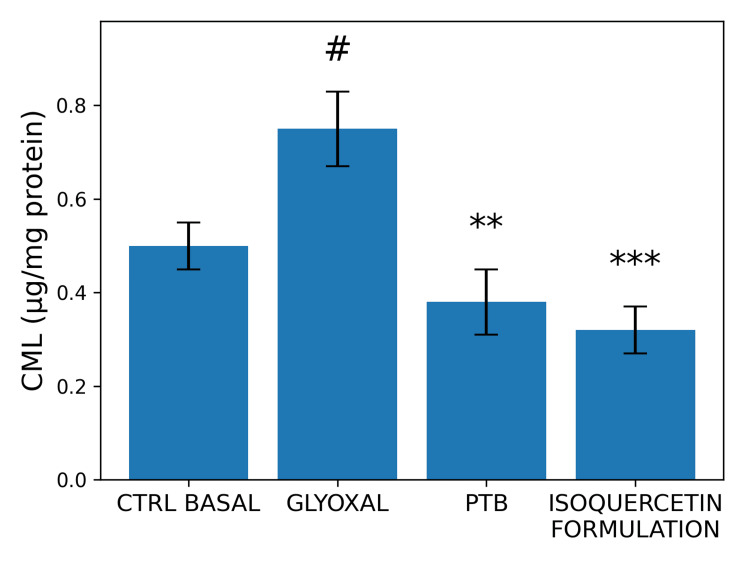
Effects of an isoquercetin-containing formulation on carboxymethyl-lysine (CML) production in human dermal fibroblast cultures subjected to glycation. Glyoxal (200 µM) was used as a glycation inducer, and N-phenacylthiazolium bromide (PTB, 100 µM) was used as a positive control. Data are expressed as mean ± standard deviation (SD) from three replicates (n = 3), according to the laboratory protocol. Statistical analysis was performed using one-way ANOVA followed by Tukey’s post hoc test. #p < 0.05 versus basal control (CTRL) group; **p < 0.002 and ***p < 0.001 versus glyoxal-treated group.

In the antiglycation protocol, pre-treatment reduced CML formation by 56.90% compared with glyoxal-treated controls (one-way ANOVA with Tukey post hoc test, p < 0.05) (Figure 8). The positive control (N-phenacylthiazolium bromide (PTB) reduced CML formation by 50.47% under the same conditions.

**Figure 9:**
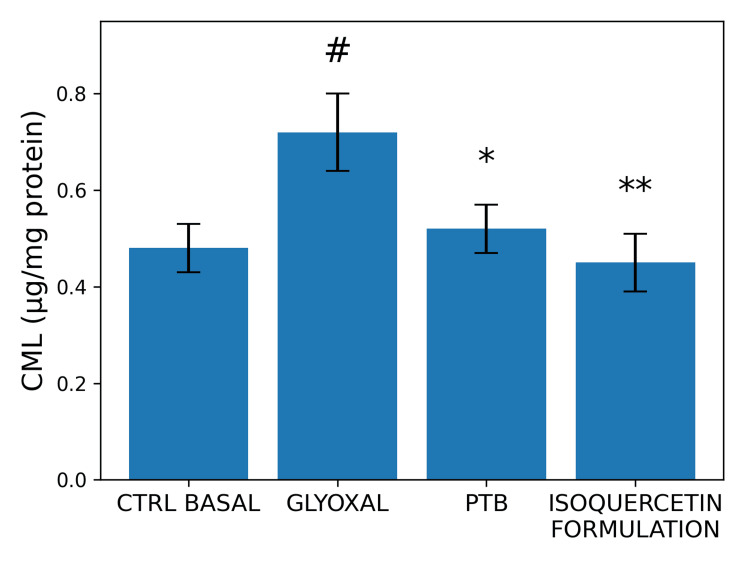
Effects of an isoquercetin-containing formulation on carboxymethyl-lysine (CML) levels in human dermal fibroblast cultures subjected to a deglycation protocol. Glyoxal (200 µM) was used to induce glycation, and N-phenacylthiazolium bromide (PTB, 100 µM) was used as a positive control. Data are expressed as mean ± standard deviation (SD) from three replicates (n = 3), according to the laboratory protocol. Statistical analysis was performed using one-way ANOVA followed by Tukey’s post hoc test. #p < 0.05 versus basal control (CTRL) group; *p < 0.05 and **p < 0.002 versus glyoxal-treated group.

In the deglycation protocol, post-treatment reduced CML levels by 39.75%, while PTB reduced CML formation by 27.99% (one-way ANOVA with Tukey post hoc test, p < 0.05) (Figure 9).

Within the defined in vitro glycation model, these findings are consistent with the modulation of advanced glycation end-product markers under controlled experimental conditions.

Collectively, the preclinical assays, experimental models, and main biological outcomes assessed in this study are summarized in Table 1.

**Table 1:** Summary of preclinical assays, experimental models, and main biological outcomes

Preclinical Assay	Experimental Model	Main Findings
NQO1 gene expression	Human skin explants	• Increased NQO1 gene expression • Enhancement of endogenous enzymatic antioxidant activity
Anti-inflammatory activity	Three-dimensional reconstructed human epidermis enriched with peripheral blood mononuclear cells (HaCaT + PBMCs)	• Reduced relative synthesis/release of IL-6 and IL-8 • In vitro anti-inflammatory effect
Oxidative stress assessment (radical status factor (RSF) and radical protection (RP))	Human skin explants	• RSF = 3.01 and RP = 66.79% • High radical/antioxidant protection
Ki-67 and type I pro-collagen immunostaining and semi-quantification	Human skin explants	• Increased Ki-67 expression → enhanced epidermal renewal • Increased type I pro-collagen expression → improved dermal support and elasticity
Carboxymethyl-lysine (CML) quantification (advanced glycation end product)	Human dermal fibroblasts (HFF-1)	• Reduced CML levels • Antiglycation activity (pre-treatment protocol) • Deglycation activity (post-treatment protocol)

## Discussion

This exploratory preclinical study evaluated the biological effects of an isoquercetin-containing formulation in validated in vitro and ex vivo models relevant to skin aging. Within the experimental conditions tested, the formulation was associated with statistically significant modulation of oxidative stress markers, inflammatory mediators, proliferative activity, collagen-related protein expression, and glycation endpoints.

The observed increase in NQO1 gene expression in human skin explants suggests activation of antioxidant-responsive pathways. NQO1 is a recognized downstream target of Nrf2 signaling and serves as a marker of endogenous cytoprotective responses against oxidative stress [10-13]. While direct assessment of Nrf2 activation was not performed, the upregulation of NQO1 may indicate engagement of cellular antioxidant defense mechanisms under the tested conditions. This transcriptional modulation was accompanied by a reduction in UV-induced protein radicalization, demonstrating concordant findings between gene expression and oxidative stress endpoints within the experimental framework employed.

In the three-dimensional reconstructed epidermal model enriched with PBMCs, treatment with the isoquercetin-containing compound was associated with reduced IL-6 and IL-8 release compared with lectin-stimulated controls. Given the recognized role of chronic low-grade inflammation in skin aging and extracellular matrix remodeling [14,15], modulation of these cytokines may reflect attenuation of inflammatory signaling pathways in this controlled experimental system. Importantly, although cytokine levels were reduced under defined laboratory conditions, these findings should be interpreted within the inherent limitations of in vitro inflammatory models.

Immunofluorescence analysis demonstrated increased Ki-67 expression and enhanced type I pro-collagen staining intensity in treated explants. Ki-67 is widely used as a marker of cellular proliferation [16,17], and its increased expression may suggest augmented epidermal renewal activity under the experimental conditions evaluated. Similarly, increased pro-collagen expression may indicate modulation of dermal matrix-related protein synthesis. However, because these findings derive from semi-quantitative immunofluorescence analysis in ex vivo explants, extrapolation to structural dermal remodeling in vivo should be approached with caution.

Glycation represents an additional pathway implicated in age-associated dermal alterations. Advanced glycation of dermal collagen has been associated with structural alterations, increased matrix rigidity, and impaired biological functionality in reconstructed skin models [18]. The observed reduction in CML levels in the present study suggests a potential modulation of glycation-related pathways under the experimental conditions evaluated. The use of reconstructed and cell-based models to investigate glycation-associated aging mechanisms has been previously validated [15,19], supporting the biological relevance of the experimental approach applied here.

Collectively, the data indicate that the isoquercetin-containing formulation was associated with multimodal biological modulation across pathways commonly implicated in skin aging, including oxidative stress regulation, inflammatory mediator modulation, extracellular matrix-related protein expression, and glycation endpoints. However, it is important to emphasize that the formulation contains multiple active ingredients, including retinol, vitamin E, hyaluronic acid, and nicotinamide. Therefore, the specific contribution of isoquercetin as an isolated component cannot be determined within the present experimental design.

Several limitations should be acknowledged. The study was conducted exclusively in in vitro and ex vivo systems with limited biological replication. Statistical reporting was based on summarized laboratory output (mean ± SEM or SD and reported p-values), and raw datasets were not available for expanded dispersion analysis or effect size calculation. Additionally, mechanistic signaling pathways were inferred based on established biological associations rather than directly quantified. Consequently, the findings should be interpreted as exploratory and hypothesis-generating rather than confirmatory.

Further investigations incorporating expanded biological replication, full raw data reporting, mechanistic pathway validation, and controlled clinical evaluation will be necessary to determine whether the observed biological modulation translates into measurable clinical outcomes in human populations.

## Conclusions

In this exploratory preclinical investigation, an isoquercetin-containing formulation was associated with statistically significant modulation of oxidative stress markers, inflammatory cytokine release, proliferative activity, collagen-related protein expression, and glycation endpoints in validated in vitro and ex vivo skin models.

Within the experimental conditions evaluated, the formulation was associated with multimodal biological modulation across pathways implicated in skin aging. However, these findings were generated in controlled preclinical systems and should be interpreted within the limitations inherent to in vitro and ex vivo experimental designs, limited biological replication, and summarized statistical reporting. Further studies incorporating expanded biological datasets, direct mechanistic validation, and controlled clinical investigations are necessary to determine whether the observed biological modulation translates into measurable and clinically meaningful outcomes in human populations.
